# IBSP Promotes Breast Cancer Bone Metastasis and Proliferation via BMP‐SMAD Signaling Pathway

**DOI:** 10.1002/cnr2.2153

**Published:** 2024-08-08

**Authors:** Wei Ding, Di Lv, Mengshen Wang, Dongsheng Pei

**Affiliations:** ^1^ Department of General Surgery Affiliated Hospital of Xuzhou Medical University Xuzhou Jiangsu China; ^2^ Department of Thyroid and Breast Surgery Affiliated Hospital of Xuzhou Medical University Xuzhou Jiangsu China

**Keywords:** breast cancer, IBSP, metastasis, proliferation

## Abstract

**Background:**

Integrin‐Binding Sialoprotein (IBSP) has been implicated in tumor progression across various cancers. However, the specific role of IBSP in breast cancer remains underexplored. There is a need to investigate the mechanisms by which IBSP influences breast cancer progression and its potential as a therapeutic target.

**Aims:**

This study aims to elucidate the role of IBSP in breast cancer, particularly its impact on tumor progression and its relationship with prognosis. We also seek to understand the underlying mechanisms, including the involvement of the BMP‐SMAD signaling pathway, and to explore the potential of targeting IBSP for therapeutic interventions.

**Methods and Results:**

Overexpression of IBSP in breast cancer cells led to increased migration and invasion, whereas IBSP interference reduced these behaviors, indicating its role in enhancing tumor progression. Differentially expressed genes were significantly enriched in the BMP‐SMAD signaling pathway, a critical pathway for osteogenic differentiation. Transcription Factor Binding: Dual luciferase reporter assays demonstrated that SMAD4 specifically binds to the IBSP promoter, establishing a regulatory link between SMAD4 and IBSP expression. Silencing IBSP (si‐IBSP) mitigated the effects of SMAD4‐induced tumor proliferation, confirming that IBSP acts as a downstream target of SMAD4 in the BMP signaling pathway.

**Conclusion:**

Our study reveals that IBSP plays a significant role in breast cancer progression through the BMP‐SMAD4 signaling pathway. Targeting IBSP could be a promising therapeutic strategy for breast cancer treatment. Further research into IBSP inhibitors may offer new avenues for improving treatment outcomes and managing breast cancer more effectively.

## 1 Introduction

Over the world, breast cancer is one of the most frequently diagnosed cancers, and is a major contributors to cancer‐related death among women [1]. Bone metastasis is the most common site of recurrence in breast cancer patients, occurring in approximately 60%–75% of cases throughout the course of the disease [2, 3]. Bone metastasis can cause a range of bone‐related diseases and seriously affected the patients' quality of life [4, 5]. Long‐distance metastasis is the dominant factor in the death of patients with breast cancer. Currently, multiple treatments are available for breast cancer, such as radiation therapy, surgery, monoclonal antibodies, antibody‐drug conjugation systems (ADCs), and immunotherapy [6], but the therapeutic effects are limited [7]. Therefore, finding effective targets to prevent and treat breast cancer is of great importance and will improve patients' quality of life and prognosis.

Integrin‐Binding Sialoprotein (IBSP), consisting of seven exons, is a member of the small integrated protein combined with the ligand N‐linked glycoprotein family that is primarily found to be expressed by bone and chondrocytes [8]. IBSP participates the coding of bone matrix and has a large influence on the formation, update, and repair of bones [9]. New evidence shows that IBSP is involved in the progress of tumors [10, 11], including accelerate matrix breakdown and promote tumor invasion [12]. IBSP upregulates in colon, breast, prostate, and lung cancers, [13] and its abnormal expression will increase the risk of bone metastasis. It has been found that IBSP can attract osteoclasts to aggregate and make bones rich in osteoclasts [14]. In addition, IBSP affects the tumor microenvironment and reduce the bone density of the lesion through receptors on the surface of the OC cells, thereby promoting bone metastases [9]. Furthermore, IBSP exerts an essential function in bone metastasis of estrogen‐positive breast cancer cells [9]. The specific upstream regulatory factor of IBSP and its implications in tumor treatment are still not clearly understood, as evidenced by limited research findings [11].

During the growth and development of vertebrates, bone morphogenetic proteins (BMPs) plays a vital role, which can induce the differentiation process of chondrocytes and bone cells in bones and improve their function [15]. BMP combined with a heterotetrameric receptor complex, which can induce SMADs phosphorylation and then could form a complex with SMAD4 to respond to BMP [16]. SMAD proteins contain 500 amino acids, comprising a large‐changing N‐terminal domain with a conservative C‐terminal domain, distributed over the entire cell, or shuttles between the nucleus and cytoplasm [17]. The BMP‐SMAD signaling pathway is a pivotal pathway involved in osteogenic differentiation [18]. SMAD4 is also called Co‐SMAD [19], which is activated by TGF‐β via serine–threonine receptor kinases phosphorylation [20]. Previous study results show that the therapeutic and prognostic aspects of breast cancer are affected by SMAD protein [19]. Nevertheless, less is known about whether IBSP participates in the progress of BMP‐SMAD mediated breast cancer tumors.

Here, we uncover the important effect and exact underlying mechanism of IBSP in breast cancer metastasis. We revealed that upstream transcription factor SMAD4 enhances the translation of downstream IBSP that can promote tumor migration and invasion. Furthermore, we found that inhibiting the SMAD‐IBSP linkage pathway in breast carcinoma can inhibit tumor cell proliferation and migration, thereby regulating cancer progression. Our finding provides novel insights into the prevention and treatment of metastasis of breast cancer.

## 2 Materials and Methods

### 2.1 Cell Culture

MDA‐MB‐231 and MCF‐7 cells were purchased from the Chinese Academy of Sciences, Chinese Cell Bank, and incubated under 5% CO_2_ at 37°C in Dulbecco's Modified Eagle's Medium (DMEM, Bio‐Channel Biotechnology) assisted with 1% penicillin–streptomycin (Vicmed) and 10% fetal bovine serum (FBS; Gibco), and changed the culture medium as often as 48–72 h.

### 2.2 Gene Transfection and Silencing

IBSP siRNA (si‐IBSP) and negative control siRNA (si‐NC) were bought from IBSBIO, and the IBSP plasmid was constructed. According to the instructions, we used the Lipofectamine 2000 reagent (Invitrogen, USA) to perform transient plasmid transfections. Following the transfection for 24–48 h, the cells were collected and verified for transfection efficiencies through western blotting.

### 2.3 Patients and Samples

Samples from six patients with metastatic breast cancer were collected and divided into a group with bone metastases and a group with metastases to other locations. The samples were sent to a biological company for RNA sequencing, and the sequencing results were analyzed using R software. According to the Declaration of Helsinki, the study protocol was approved by the Ethics Committee of the Affiliated Hospital of Xuzhou Medical University (XYFY2022‐KL405‐01). Clinical specimens were obtained with the informed consent of patients.

### 2.4 Western Blot Analysis

Total sample of cells was gathered and followed with phenylmethanesulfonyl fluoride (Vicmed), a kind of proteinase inhibitor, supplemented with RIPA lysis buffer (Vicmed) to lyse them. Protein concentration was assayed using a protein detection kit (Beyotime). On 10% SDS–PAGE gels, protein samples were separated through electrophoresis followed by transfer to nitrocellulose membranes. The films were blocking with 5% skimmed milk for at least 2 h before incubation overnight with the primary antibodies at a temperature of 4°C. Membranes were then incubated for 2 h using the secondary antibodies after washing in PBS. Blots were detected by an augmented chemiluminescent substrate reagent (Tanon). Following primary and second antibodies were utilized: anti‐snail1 (A5243, ABclonal), anti‐E‐cadherin (A3044, ABclonal), anti‐N‐cadherin (A3045, ABclonal), anti‐Flag (0912‐1, HUABIO), anti‐SMAD4 (10231‐1‐AP, Proteintech), anti‐IBSP (A16220, ABclonal), anti‐Vimentin (201 158, ZENBIO), anti‐β‐actin (AC004, ABclonal), HRP‐linked anti‐rabbit secondary antibody (RGAR001, Proteintech).

### 2.5 Colony Formation Assay

MCF‐7 and MDA‐MB‐231 cells were seeded with the concentration of 500 cells per well in 6‐well plates, every well was added 1 mL the culture medium that replaced every 3 days, and the cells were placed in 5% CO_2_ and at 37°C for 14 days as to develop colonies. Fixed for 15 min with multi‐poly‐aldehyde, and then stained for 15 min by 0.1% crystalline purple, the colony formation was examined, counted, and photographed in an inverted phase contrast microscope.

### 2.6 CCK8 Assay

The vigor of cells was assessed by using the Cell Counting Kit‐8 assay (Dojindo). MCF‐7 and MDA‐MB‐231 cells were plated with a density of 1 × 10^4^ cells per well within a 96‐well plate. After 24, 48, and 72 h, the corresponding wells were respectively added 10 μL CCK‐8 reagent and co‐inflated in 37°C over a period of 4 h. Finally, the value of optical densitometry was recorded on a microtiter plate reader with a wavelength of 450 nm.

### 2.7 Scratch Wound Assay

MCF‐7 and MDA‐MB‐231 cells were planted into 6‐well plates at 1 × 10^6^ cells per well. Over‐expression and interference plasmid were, respectively, expressed in MCF‐7 and MDA‐MB‐231 cells. Adding over‐expression plasmid for 24 h and interference plasmid for 48 h later, we used the 100 μL gun tips to quickly draw a straight line on the bottom surface, and immediately replaced the solution to serum‐free medium and took pictures under the electronic microscope, another one was taken in the same position in 48 h.

### 2.8 Transwell Invasion and Migration Assay

The capacity of MCF‐7 and MDA‐MB‐231 cells to migrate and invade was examined with cell migration and transwell assays transwell chambers (BD Bioscience). MCF‐7 cells and MDA‐MB‐231 cells were cultured in 150 μL of the serum‐free medium then planted into the superior chamber, along with 600 μL DMEM and 10% fetal bovine serum filled into the inferior chamber. The day before invasion test, 20 μL of Matrigel (BD Bioscience) was placed inside the upper chambers. After incubated for 24 h, cells at the interior bottom of the superior lumen were carefully wiped away, cells crossed the bottom membrane were immobilized in 4% paraformaldehyde with 15 min, carried on dyeing with 0.1% crystal violet with another 15 min. At last, we used an electronic microscope for image collection and counting.

### 2.9 RNA Isolation and Real Time Quantitative Reverse Transcription PCR

TRIzol reagent (Vazyme, Nanjing, China) was applied to harvest gross RNA and the SweScript RT I First Strand cDNA Synthesis Kit (Servicebio) was utilized to perform cDNA synthesis. By running the SYBR Green qPCR Master Mix (High ROX) (Servicebio) and 2^−△△Ct^ method on an ABI StepOne Plus RealTime PCR System, the results of cDNA were visualized by quantitative RT‐PCR (qPCR).

### 2.10 Chromatin Immunoprecipitation Assay

MCF‐7 and MDA‐MB‐231 cells were cross‐linked for 10 min at ambient temperature with formaldehyde (1%), then mixed in glycine to halt the interaction for 5 min. Cells were collected with PBS containing bright peptone, aprotinin, and PMSF, then the DNA fragments were sheared into 150–300 bp by using ultrasonic. After the centrifugation, the upper liquid was divided into three copies, one was stored at −80°C and the other two were respectively added primary antibodies, anti‐IgG antibody. Next, adding immune magnetic beads into the crack antibody and incubating for 90 min at 4°C with rotation. We employed low‐immune complex, high‐immune complex, LiCl immune, and TE buffer to wash and capture the protein/DNA complexes, using 10% Chelex‐100 and protease K to separate immune precipitated DNA and that was purified with rotating columns. Finally, PCR or QPCR was applied to amplify the targeted DNA.

### 2.11 Promoter Cloning and Generation of Dual‐Luciferase Reporter Assay

The pGL3 basic vector (Promega, USA) was used to clone and insert a human IBSP promoter spanning from −2000 to +200 bp. Subsequently, deletion experiments were conducted to generate reporter plasmids spanning from −400 to +200 bp. Mutant IBSP reporter plasmids were created based on the −2000/+200 bp plasmid. MCF‐7 cells were co‐transfected with luciferase reporter plasmids as well as SMAD4 plasmid. Up to 24 h after transfection, cells were harvested. 48 h after transfection, the relative firefly luciferase activity was determined using a Dual‐Luciferase Reporter Assay System (Promega) and standardized with those of Renilla luciferase.

### 2.12 Kaplan Meier‐Plotter Analysis

Kaplan Meier Plotter could assess the effects of 54K genes on survivorship in 21 cancers. The sources for its database include EGA, GEO, and TCGA. Survival analysis is a commonly used analysis method in biomedical research. In the queue follow‐up research, we will define some observation endpoints in advance, which are called events. The time interval from the beginning to the incident from research to the incident. The log‐rank test is a commonly used non‐parameter method for survival comparison between groups. We evaluated prognosis for IBSP among patients suffering from breast cancer.

### 2.13 GEPIA2 Database Analysis

Gene expression profiling interactive analysis (GEPIA) is a dynamic analysis of gene expression profile, and its data are derived from Genotype‐Tissue Expression (GTEX) and The Cancer Genome Atlas Program (TCGA) Database. It can analyze the RNA sequencing data from 8587 normal samples and 9736 tumor samples in GTEX and TCGA programs. The differential analysis results of GEPIA2 are default to compare the tumor tissue of TCGA with the normal tissue of GTEX, which is used for analysis of the expression spectrum and interactive analysis of cancer and normal genes, promoting the development of cancer genome research.

### 2.14 GO Analysis and Gene Set Enrichment Analysis

The GO analysis of IBSP and SMAD4 were determined using R software (version: 4.3.2), where the “clusterprofiler” package was used to print curve plots. *p* value <0.05 was regarded as statistically meaningful.

### 2.15 Statistical Analysis

As for all statistical analyses, SPSS 16.0 was performed and GraphPad Prism 9.0 was applied for images. All values were confirmed through three separate repeated trials and presented in mean value ± standard deviation (SD). We utilized *T* tests to compare normally distributed variables between two groups, while employing one‐way ANOVA for statistical comparisons involving more than three groups. Bonferroni post hoc test was used after ANOVA. *p* value <0.05 (*), *p* value <0.01 (**), or *p* value <0.001 (***) was considered statistically significant.

## 3 Results

### 3.1 IBSP Predicts Poor Prognosis of Breast Cancer

**FIGURE 1:**
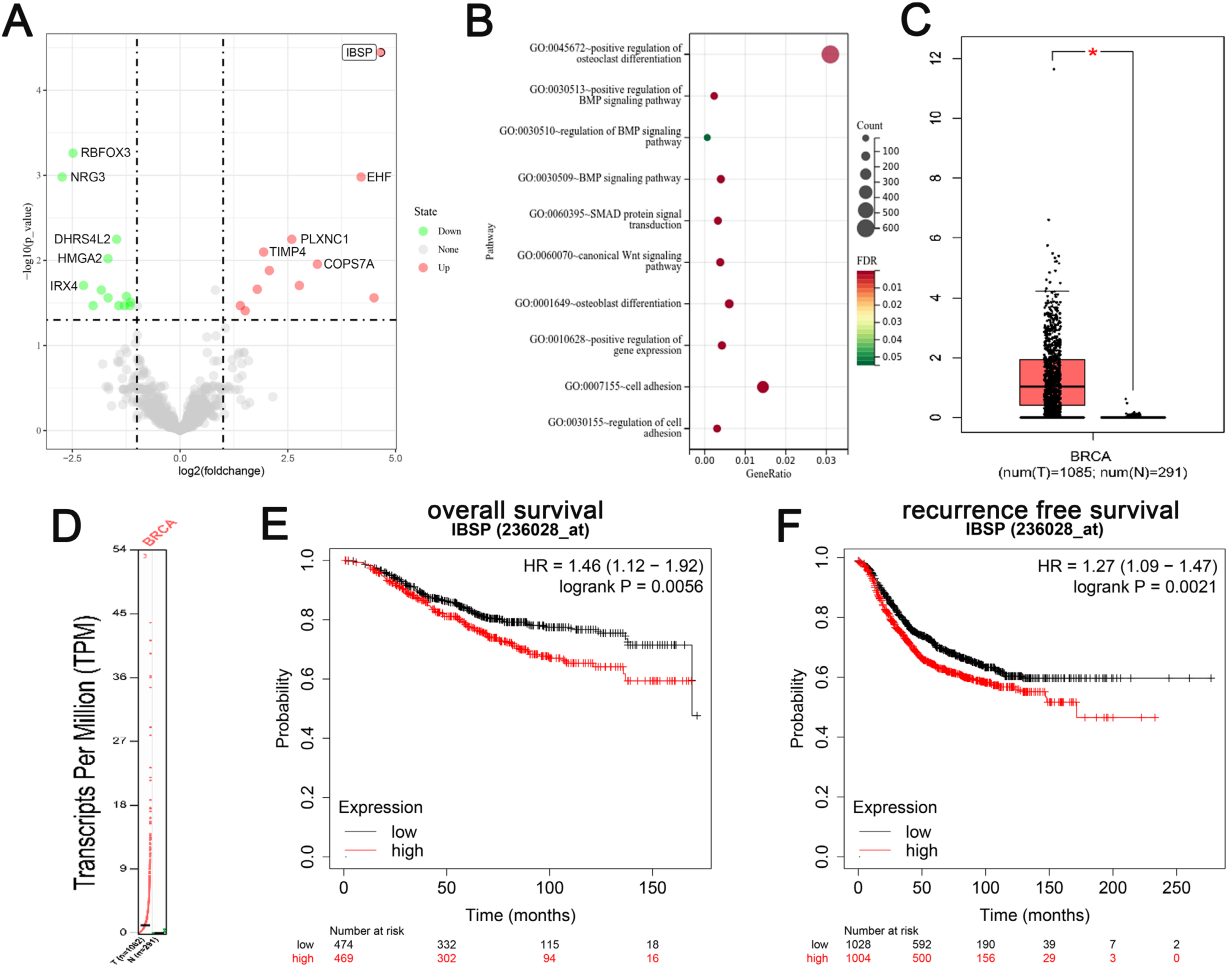
IBSP was strongly associated with breast cancer progression. (A) The expression of IBSP in bone metastasis samples was detected by RNA sequencing. (B) Enrichment pathways for the differentially expressed genes were revealed using GO enrichment analysis. (C and D) The GEPIA2 database showed that IBSP was expressed at high levels in breast cancer. (E and F) The Kaplan–Meier survival curves analyzed the association of IBSP expression with overall survival and recurrence free survival in breast cancer samples.

To investigate whether IBSP has a significant effect in breast carcinoma cell bone metastasis, we performed RNA sequencing and found that IBSP expression significantly increased in bone metastasis samples (Figure 1A). Further, GO enrichment assay suggested that the differential expression genes prominently enriched in the BMP‐SMAD pathway and osteoclast differentiation pathway (Figure 1C). Bioinformatics analysis from GEPIA2 database indicated that IBSP was clearly elevated in breast cancer tissue samples (Figure 1D,E), and the high expression of IBSP notably reduced general and progress free survival of patients (Figure 1F,G). These findings demonstrated that IBSP has great potential to perform an influential part in the therapy of breast cancer patients.

### 3.2 IBSP Promotes Breast Cancer Progression

**FIGURE 2:**
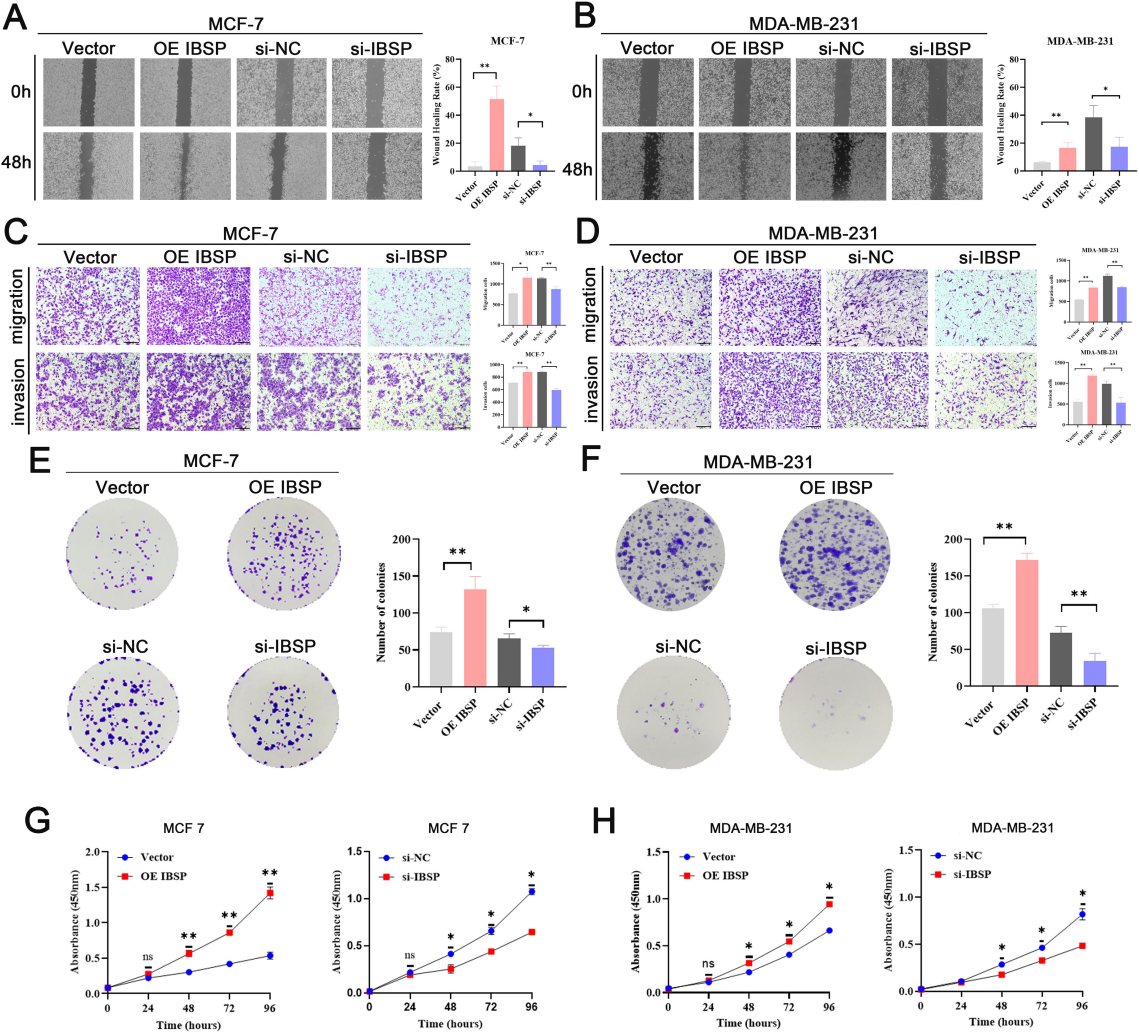
IBSP promoted cell migration and invasion. (A and B) The cell motility of MCF‐7 (A) and MDA‐MB‐231 (B) cells transfected with IBSP or knockdown IBSP were detected by wound healing test. (C and D) Transwell assays were performed to depict the cell migration and invasion in MCF‐7 (C) and MDA‐MB‐231 (D) cells after treatment with overexpression IBSP or si‐IBSP. (E and F) Colony formation assays were performed in MCF‐7 (E) and MDA‐MB‐231 (F) cells after transfection with IBSP overexpression or interference to detect the proliferation capacity of cell. (G and H) Cell reproduction in MCF‐7 (G) and MDA‐MB‐231 (H) cells after overexpression or knockdown IBSP were detected by CCK‐8 experiments (*N* = 3, *p < 0.05, **p < 0.01, ***p < 0.001).

To explore the relationship between IBSP and breast cancer progression, wound healing test was performed and results showed that IBSP overexpression enhanced the ability of cell motility, whereas IBSP knock down showed the adverse effect (Figure 2A,B). Transwell migration/invasion assays manifested that IBSP obviously accelerated the capacity of cell migration and invasion, which was restrained by knocking down IBSP (Figure 2C,D). Colony formation experiments demonstrated that over‐expressing IBSP boosted cell proliferation, whereas si‐IBSP suppressed the capacity of cell proliferation (Figure 2E,F). CCK‐8 experiments demonstrated that IBSP stimulate cell reproduction, si‐IBSP notably inhibited cell reproduction (Figure 2G,H). Taken together, our results suggested that IBSP facilitates breast cancer migration and invasion.

### 3.3 IBSP and SMAD4 Are Positively Correlated With Cell Proliferation and Cell Adhesion

**FIGURE 3:**
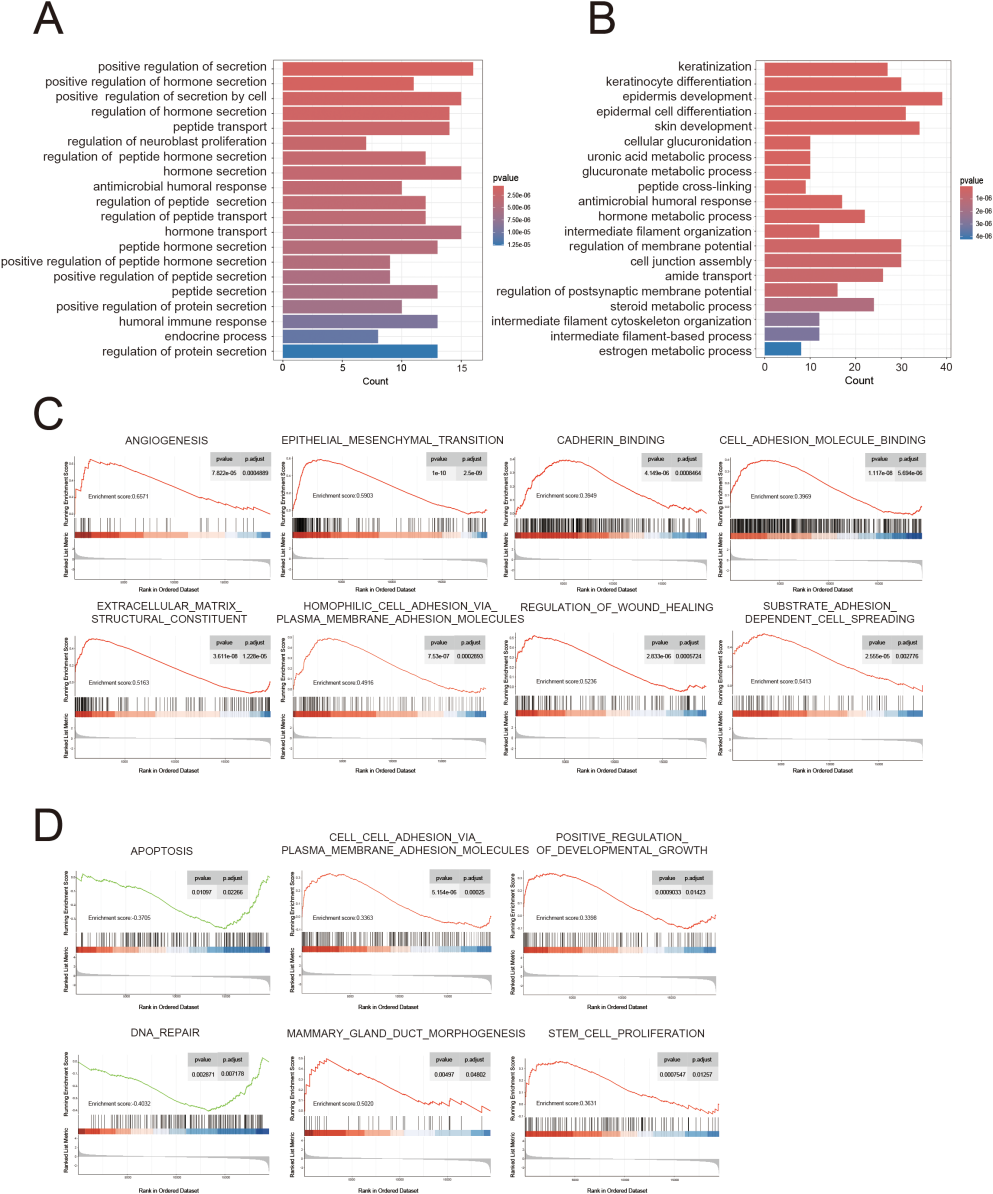
IBSP and SMAD4 are positively correlated with cell proliferation and cell adhesion. (A) The GO enrichment analysis of IBSP. (B) The GO enrichment analysis of SMAD4. (C) The GSEA analysis of the expression of IBSP. (D) The GSEA analysis of the expression of SMAD4.

GO analysis show the enrichment of IBSP and SMAD4 (Figure 3A,B). GESA analysis show that the angiogenesis, epithelial mesenchymal transition, cadherin conjugation, cell adherence molecule attachment, extracellular matrix structural components, homophilic cellular attachment through plasma membrane adhesion, wound healing regulation, and substrate adhesion dependent cell spreading were enriched in the group of high expression of IBSP (Figure 3C). And the apoptosis and DNA repair were enriched in the low expression of SMAD4. What is more, the cell adhesion through plasmalemmal adhesion polymers, mammary gland duct morbidity, development and growth, and stem cell proliferation were enriched in high expression of SMAD4 (Figure 3D). Additionally, Figure S1 showed the KEGG analyses of IBSP and SMAD4. In summary, these results indicated that IBSP and SMAD4 are positively correlated with cell proliferation and cell adhesion.

### 3.4 SMAD4 Promotes Breast Cancer Progression

**FIGURE 4:**
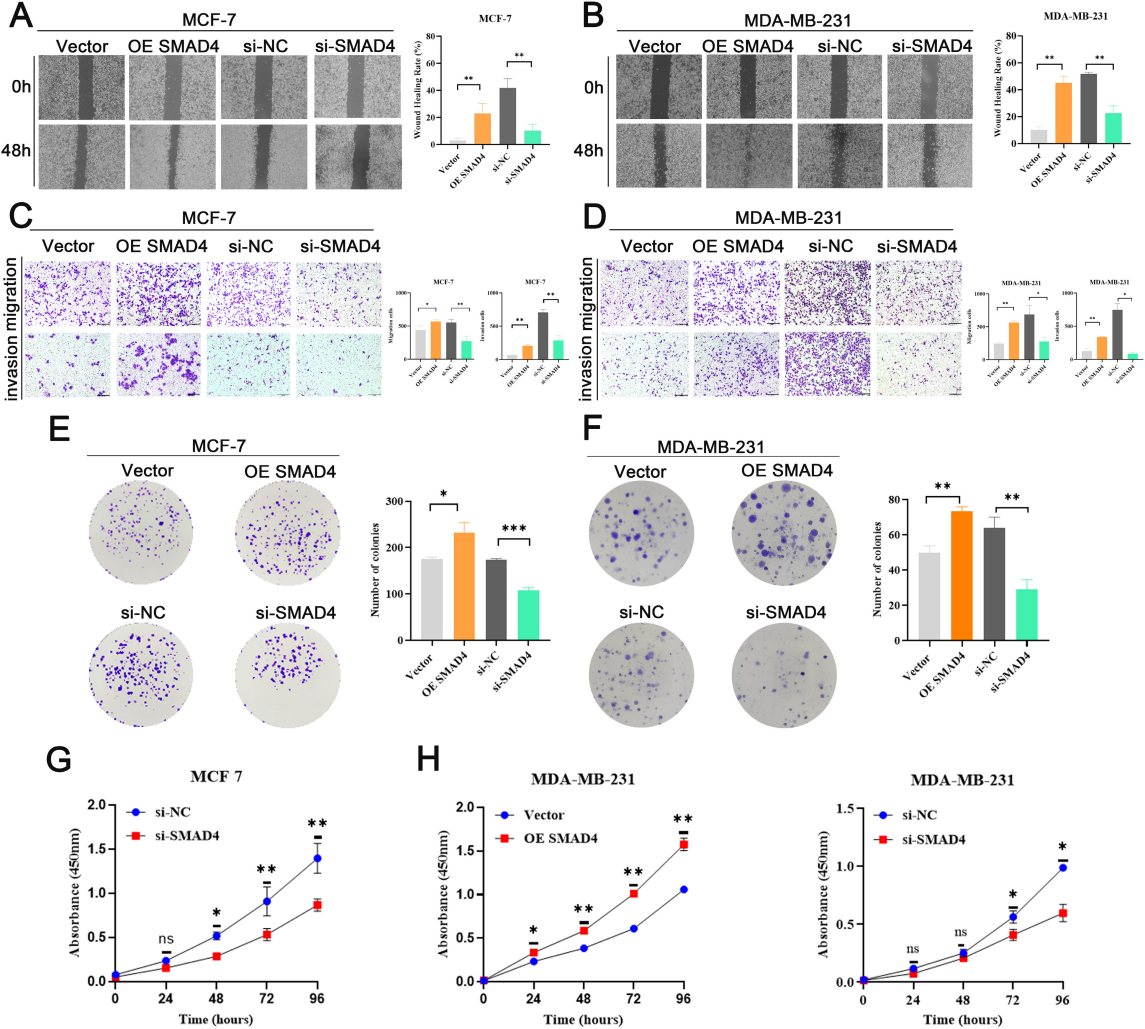
SMAD4 facilitated cell migration and invasion. (A and B) Wound healing test was performed to depict the cell motility of MCF‐7 (A) and MDA‐MB‐231 (B) cells after overexpression or knockdown SMAD4. (C and D) The cell migration and invasion in MCF‐7 (C) and MDA‐MB‐231 (D) cells after transfection with SMAD4 overexpression or interference were detected by transwell assays. (E and F) Colony formation experiment was performed in MCF‐7 (E) and MDA‐MB‐231 (F) cells after treatment with overexpression SMAD4 or si‐SMAD4 to detect the cell proliferation capacity. (G and H) Cell reproduction in MCF‐7 (G) and MDA‐MB‐231 (H) cells transfected with SMAD4 or knockdown SMAD4 were detected by CCK‐8 experiments (*N* = 3, *p < 0.05, **p < 0.01, ***p < 0.001).

BMP connected with a receptor complex, which can guide SMADs phosphorylation and then form a complex with SMAD4 to respond to BMP [16]. Next, we explored the link between SMAD4 and breast cancer progression. Would healing assays indicated that SMAD4 contributes to cell motility, which showed as restrained when SMAD4 was knocked down (Figure 4A,B). Transwell tests were carried out and results manifested overexpression of SMAD4 facilitates cell migration and invasion, yet SMAD4 knock down displayed the opposite effect (Figure 4C,D). To illustrate cell proliferation, we carried out colony formation assays, and it can be found that SMAD4 stimulated cell proliferation, whereas si‐SMAD4 significantly inhibited it (Figure 4E,F). CCK‐8 assays showed that cell reproduction could be curbed by knockdown of SMAD4 and be promoted by overexpression of SMAD4 (Figure 4G,H). In conclusion, our analyses suggested that SMAD4 enhances tumor migration and invasion, and may be closely linked to tumor progression for breast cancer patients.

### 3.5 SMAD4 Binds IBSP Promoter and Promote Transcription

**FIGURE 5:**
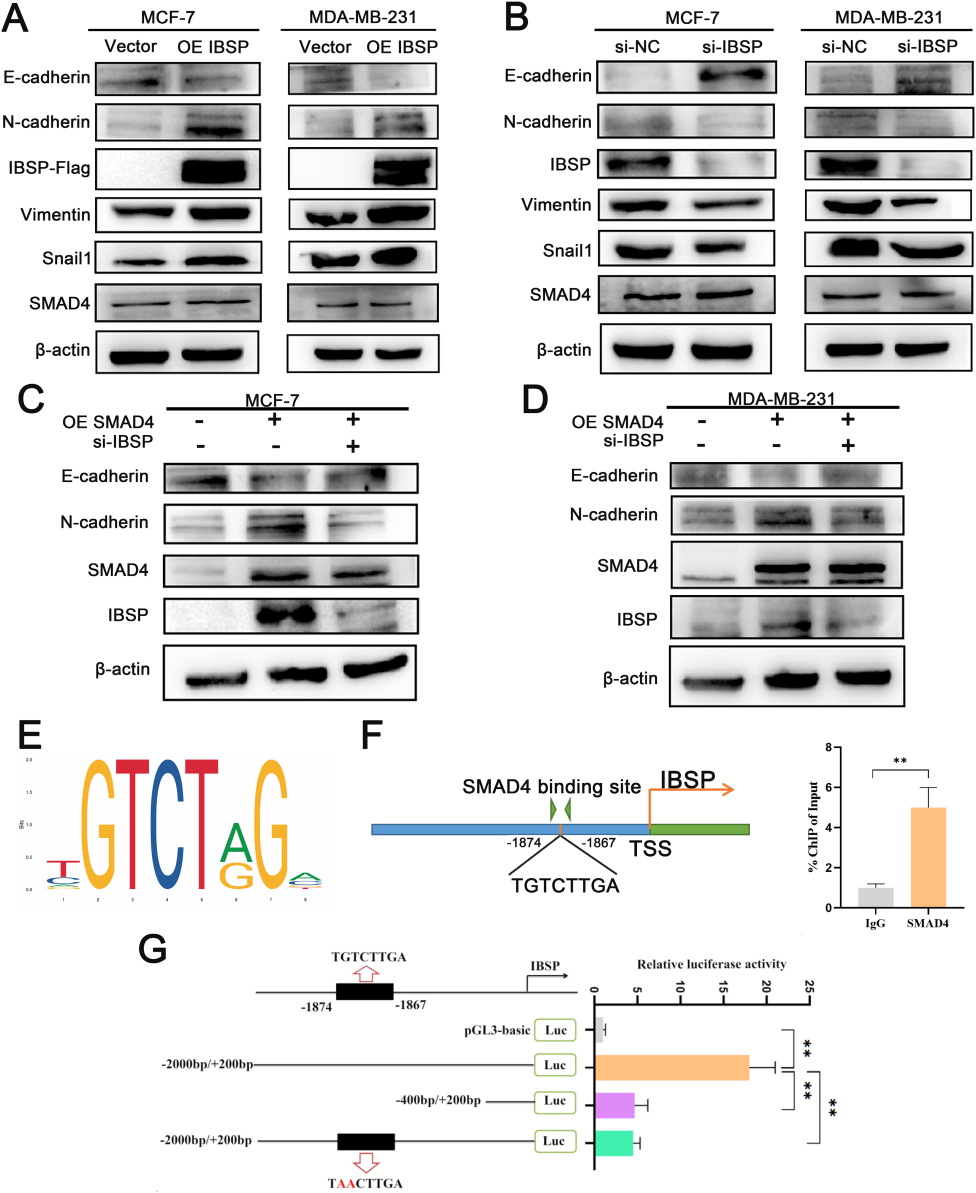
The transcription factor SMAD4 binds specifically to the IBSP promoter. (A and B) Immunoblotting analysis of E‐cadherin, N‐cadherin, vimentin, snail1, and β‐actin in MCF‐7 and MDA‐MB‐231 cells with overexpression IBSP (A) and knockdown IBSP (B). (C and D) Immunoblotting experiments were performed to analyze the levels of E‐cadherin, N‐cadherin, SMAD4, IBSP, and β‐actin in MCF‐7 and MDA‐MB‐231 cells co‐transfected with SMAD4 overexpression (C) and si‐IBSP (D). (E) The transcription factor motif enrichment analysis of SMAD4. (F) The JASPAR database was utilized to forecast the specific binding site of SMAD4 in the IBSP promoter, and CHIP assays were carried out for validation (**p < 0.01). (G) Dual‐luciferase reporter assays was performed to verify the effective binding region of IBSP to SMAD4, deletion and mutation in the SMAD4 motif (GT to AA) were indicated.

Previous studies suggested that epithelial mesenchymal transition (EMT) stimulated migration and invasion of tumor cells exerts a powerful effect on the process of occurrence, development, invasion metastasis, and drug resistance [21, 22, 23]. Immunoblotting analysis indicated that overexpressing IBSP in breast carcinoma cells significantly increased expression levels of genes such as vimentin, N‐cadherin, and snail1, and down‐regulates E‐cadherin expression, which promoted EMT and enhanced cell metastasis. On the contrary, interfering IBSP had the opposite effect, inhibiting the expressed levels of these genes and suppressing tumoral progression (Figure 5A,B). For exploring whether SMAD4 regulates the aggression and metastasis of breast cancer cells via IBSP, we performed rescue experiments in which cells were co‐transfected with SMAD4 and si‐IBSP. The results showed that interfering with IBSP restored the expression levels of N‐cadherin, vimentin, and snail1, and the level of cell motility, up‐regulated the level of E‐cadherin, suggesting that SMAD4 modulates breast cancer migration and invasion through IBSP (Figure 5C,D). Consequently, for the purpose of further exploring the effect of SMAD4 in the regulation of IBSP, elaborate promoter analyses were carried out to further investigate the regulatory mechanisms of IBSP expression. The motif of SMAD4 was obtained by using the JASPAR website (Figure 5E). Based on the SMAD4 motif sequence, we predicted a potential binding site for SMAD4 in the IBSP promoter region, located in the sequence −1874 to −1867. For the candidate binding sites, we performed ChIP experiments and observed a direct binding relationship between promoter and SMAD4 (Figure 5F). To further increase the reliability of our conclusions, we further performed dual‐luciferase reporter assays to verify the promoter activity of candidate binding sites and effective transcriptional activation sites, and the results showed the transcription factor SMAD4 binds exclusively with the IBSP promoter. When this predicted binding region was deleted or mutated, the binding ability was significantly reduced. The above results suggest that the site of IBSP binding by SMAD4 is the −1874 to −1867 sequence (Figure 5G).

### 3.6 SMAD4 Promote Breast Cancer Progression via IBSP

**FIGURE 6:**
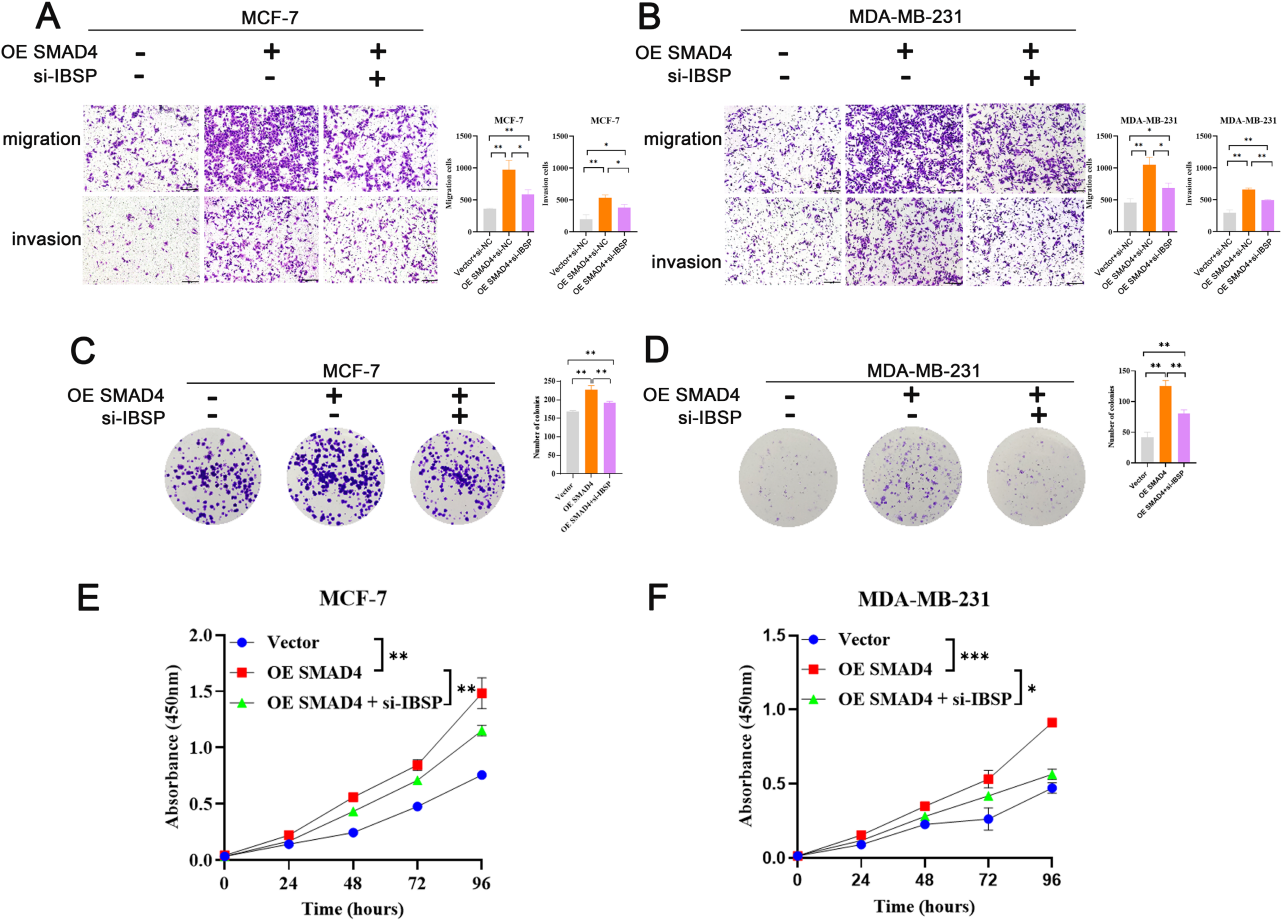
SMAD4 promoted the breast cancer migration and invasion through IBSP. (A and B) Transwell experiment images depicting changes in migration and invasion in the overexpression of SMAD4 and knockdown IBSP MCF‐7 (A) and MDA‐MB‐231 (B) cells. (C–F) Typical images of colony formation assay and CCK‐8 assay described cell proliferation in MCF‐7 (C and E) and MDA‐MB‐231 (D and F) cells co‐transfected with SMAD4 and si‐IBSP.

To further investigate the correlation between IBSP and SMAD4 in breast cancer, following experiments were carried out. Si‐IBSP could rescue cell migration and invasion induced by SMAD4 (Figure 6A,B). Knockdown of IBSP cells suppressed the SMAD4 induced growth of tumor cells (Figure 6C,D). Cell vitality was detected by the CCK8 assays, IBSP depletion markedly inhibited the cell proliferation of SMAD4‐restored cells (Figure 6E,F). Consequently, our findings implied that SMAD4 promoted the breast cancer migration and invasion through IBSP.

## 4 Discussion

Breast cancer is considered as the leading malignant tumor in women, with more than half patients developing bone metastases in advanced stages [5, 24]. The prognosis and survival time of breast cancer patients are closely related to clinical factors such as cell proliferation and tumor metastasis [6]. Therefore, finding effective targets for both preempting and treating cancer cell proliferation and osseous metastasis is crucial for improving the quality of patient survival and prognosis. For this purpose, our study screened IBSP as prospective therapeutic candidates in breast cancer. RNA sequencing implied that IBSP expression was markedly higher in bone metastasis samples. Thus, we recognized IBSP to be a promising therapeutic target with highly expressed in breast cancer bone metastasis samples. IBSP, a non‐collagenous glycoprotein involved in cell adherence, proliferation, and differentiation, has been discovered strongly correlated with the progression of tumors and promotes bone metastases by altering bone composition. IBSP is associated with cancer stem cells, which are thought to be contributors to cancer recurrence [10], whereas there are insufficient findings to show its specific regulatory mechanisms in breast cancer. GO enrichment assay demonstrated that the differential expression genes were mainly enriched in osteoclast differentiation pathway and BMP‐SMAD modeling pathway. BMP is part of the transforming growth factor (TGF)‐β superfamily, and the typical signaling pathway associated with it is the BMP‐SMAD signaling pathway, which has been shown strongly related to osteogenic differentiation.

Here, we delved into the associations in between IBSP and breast cancer progression, along with the regulation of IBSP by SMAD4, which was associated with breast cancer metastasis. Our results indicate that IBSP is overexpressed among breast cancer bone metastatic samples and its expression is accompanied by a poorer prognosis. Small interfering RNA (siRNA), sometimes called short interfering RNAs or silencing RNAs, are a class of double‐stranded RNA molecules, 20–24 base pairs in length, similar to miRNAs and operating within the RNA interference (RNAi) pathway. It interferes with the posttranscriptional degradation of mRNAs from specific genes expressing nucleotide sequences complementary to those of miRNAs, thereby preventing translation. Since in principle any gene can be knocked down by a synthetic siRNA with a complementary sequence, siRNA is an important tool for validating gene function and drug targeting [25]. In previous studies, several articles have used small interfering RNAs for cellular phenotype reversal experiments and obtained convincing results [26, 27, 28]. Further functional researches confirmed the action of IBSP within the promotion of breast cancer metastasis, as IBSP overexpression accelerated wound healing, cell migration and invasion, whereas IBSP knock down exhibits the opposite phenomenon, and emphasized the importance of IBSP as an important regulator of metastasis. Based on RNA sequencing analysis, we further performed transwell, wound healing, and CCK8 experiments and found that SMAD4 promotes the metastatic and proliferative capacity of tumors. IBSP may act differently on different types of breast cancer cells. MDA‐MB‐231 belongs to a highly metastatic malignant breast cancer cell line, MCF‐7 is an in situ ER‐positive breast cancer cell line. MDA‐MB‐231 cells are usually more invasive/metastatic than MCF‐7 cells, but in our experiments, we found that IBSP had a greater effect on MCF‐7. We guess it is because of the possibility of stimulation of certain hormones, which leads to the more invasive nature of MCF‐7, but the exact reason needs further in‐depth study. The activation of EMT is a pivotal procedure for cancer cell metastasis, and it can be determined by the expression of snail1, markers of the epithelium such as E‐cadherin and markers of mesenchymal cell for instance N‐cadherin and vimentin. EMT is characterized by the absence of E‐cadherin and upregulation of N‐cadherin, vimentin, and snail1 expression. Therefore, we verified the correlation between IBSP, SMAD4, and changes in the expression of EMT‐related markers by immunoblotting. The experimental findings suggested that when overexpressing IBSP or SMAD4, the protein expression of vimentin, N‐cadherin, and snail1 was greatly promoted, and the E‐cadherin expression was suppressed. When further interfering IBSP, the opposite effect will be exhibited, which inhibited EMT and rescued the formation of aggressive phenotype during cancer metastasis. To probe further the precise mechanism through which IBSP modulates the SMAD signaling pathway, the motif of SMAD4 were predicted by the JASPAR website, and we succeeded in identifying binding sites located in the −1874 to −1867 sequence. By constructing a dual‐luciferase reporter plasmid with a mutation in the SMAD4 predicted sites, the promoter activity of the predicted binding sequences and the effective transcriptional activation site were verified using the dual‐luciferase reporter assay, and deletion or mutation at the predictive sites will result in the IBSP failing to bind specifically to SMAD4. Transwell, colony formation, and CCK‐8 assays were further carried out to verify the relationship between IBSP and SMAD4 in tumor cells. Our results show that si‐IBSP could rescue SMAD4‐induced cell metastasis, proliferation, and inhibit tumor progression, which contributes to the treatment and prognosis of patients.

Most prior studies have only suggested a correlation between IBSP and mammary cancer [12], but the specific regulatory modality has not been addressed, and our study verified that IBSP is hyper‐expressed in breast cancer with involvement into BMP‐SMAD‐mediated bone metastases pathway in breast carcinoma, when interfering with IBSP will rescue BMP‐SMAD‐promoted tumor progression, suggesting that IBSP could be used as a potential molecular marker to assess breast cancer risk. Nevertheless, our study still has certain limitations, which could be further developed by probing the detailed mechanisms and sustained by animal testing. It is hoped that our study on the mechanism of IBSP regulation by SMAD4 will create new insights and opportunities for the therapy of breast cancer.

## Author Contributions

**Wei Ding:** conceptualization, supervision. **Di Lv:** investigation, writing – original draft. **Mengshen Wang:** investigation, writing – original draft. **Dongsheng Pei:** supervision, validation.

## Conflicts of Interest

The authors declare no conflicts of interests.

## Supporting information


**FigureS1.** KEGG analysis of IBSP and SMAD4. (A) The KEGG analysis of IBSP in breast cancer. (B) The KEGG analysis of SMAD4 in breast cancer.

## Data Availability

The data that support the findings of this study are available from the corresponding author upon reasonable request.
