# Supplementary figures and images for: IBSP Promotes Breast Cancer Bone Metastasis and Proliferation via BMP‐SMAD Signaling Pathway
Source: Cancer Rep (Hoboken). 2024 Aug 8;7(8):e2153. doi: 10.1002/cnr2.2153 (PMC11310091; doi:10.1002/cnr2.2153)

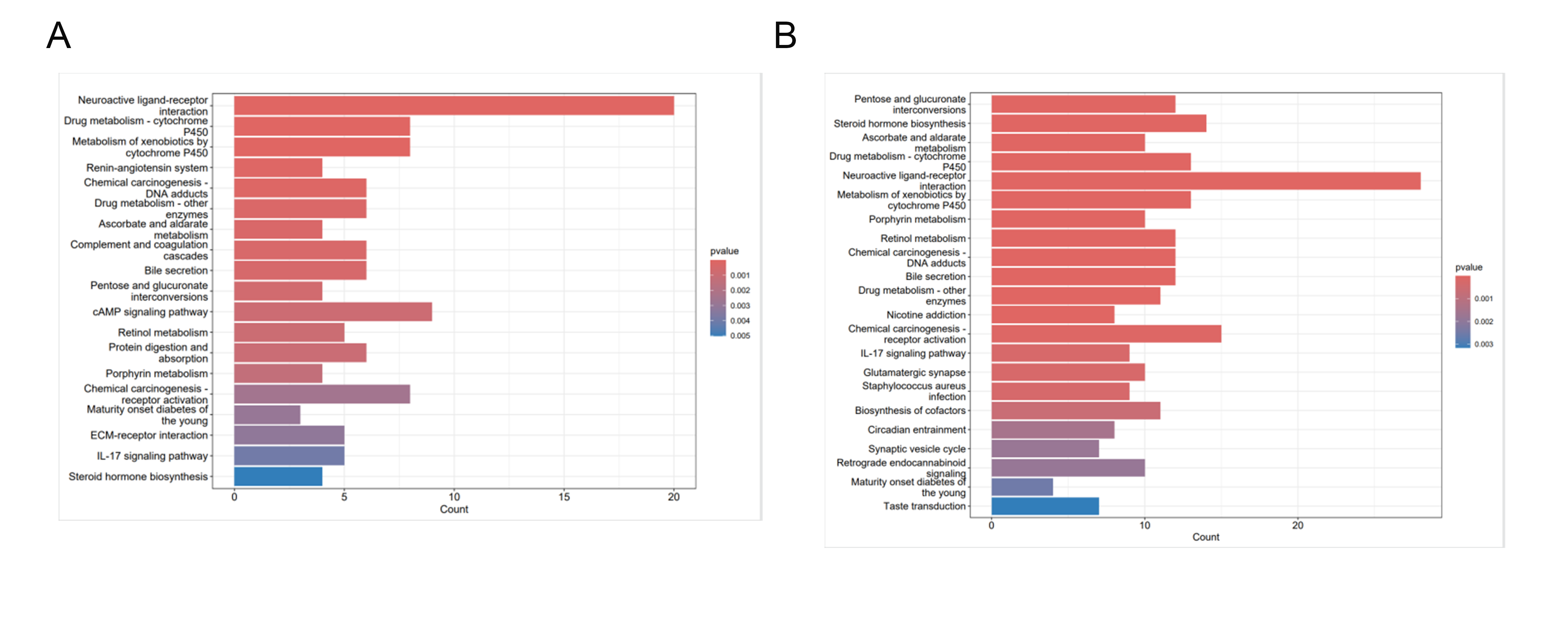

Supplement: Supplementary file 1 — FigureS1. KEGG analysis of IBSP and SMAD4. (A) The KEGG analysis of IBSP in breast cancer. (B) The KEGG analysis of SMAD4 in breast cancer. [file CNR2-7-e2153-s001.tif]
